# Mechanisms of Gastroprotective Effects of Ethanolic Leaf Extract of *Jasminum sambac* against HCl/Ethanol-Induced Gastric Mucosal Injury in Rats

**DOI:** 10.1155/2012/786426

**Published:** 2012-04-05

**Authors:** Ahmed S. AlRashdi, Suzy M. Salama, Salim S. Alkiyumi, Mahmood A. Abdulla, A. Hamid A. Hadi, Siddig I. Abdelwahab, Manal M. Taha, Jamal Hussiani, Nur Asykin

**Affiliations:** ^1^Department of Molecular Medicine, Faculty of Medicine, University of Malaya, 50603 Kuala Lumpur, Malaysia; ^2^Department of Chemistry, Faculty of Science, University of Malaya, 50603 Kuala Lumpur, Malaysia; ^3^Department of Pharmacy, Faculty of Medicine, University of Malaya, 50603 Kuala Lumpur, Malaysia; ^4^Department of Medical Microbiology, Faculty of Medicine, University Technology, MARA, 40450 Shah Alam, Malaysia

## Abstract

*Jasminum sambac* is used in folk medicine as the treatment of many diseases. The aim of the present investigation is to evaluate the gastroprotective effects of ethanolic extracts of *J. sambac* leaves against acidified ethanol-induced gastric ulcers in rats. Seven groups of rats were orally pre-treated with carboxymethylcellulose (CMC) as normal group, CMC as ulcer group, 20 mg/kg of omeprazole as positive group, 62.5, 125, 250, and 500 mg/kg of extract as the experimental groups, respectively. An hour later, CMC was given orally to normal group and acidified ethanol solution was given orally to the ulcer control, positive control, and the experimental groups. The rats were sacrificed after an hour later. Acidity of gastric content, the gastric wall mucus, ulcer areas, and histology and immunohistochemistry of the gastric wall were assessed. Gastric homogenates were determined for prostaglandin E_2_ (PGE_2_), superoxide dismutase (SOD), andmalondialdehyde (MDA) content. Ulcer group exhibited significantly severe mucosal injury as compared with omeprazole or extract which shows significant protection towards gastric mucosal injury the plant promotes ulcer protection as it shows significant reduction of ulcer area grossly, and histology showed marked reduction of edema and leucocytes infiltration of submucosal layer compared with ulcer group. Immunohistochemistry showed overexpression of Hsp70 protein and downexpression of Bax protein in rats pretreated with extract. Significant increased in the pH, mucus of gastric content and high levels of PGE_2_, SOD and reduced amount of MDA was observed.

## 1. Introduction

Peptic ulcer is a common disorder of the stomach and duodenum [1]. The basic physiopathological of gastric ulcer results from an imbalance between some endogenous aggressive factor(s) [hydrochloric acid, pepsin, refluxed bile, leukotrienes, reactive oxygen species (ROS)] and cytoprotective factors, which include the function of the mucus-bicarbonate barrier, surface active phospholipids, prostaglandins (PGs), mucosal blood flow, cell renewal and migration, nonenzymatic and enzymatic antioxidants, and some growth factors [2, 3]. The multifactorial pathogenesis of peptic ulcers is secretion of gastric acid. Therefore, the main therapeutic target is the control of this secretion using antacids, H2 receptor blockers (ranitidine, famotidine) or proton pump blockers (omeprazole and lansoprazole) [4]. However, nowadays, gastric ulcer therapy faces a major drawback because most of the drugs currently available in the market show limited efficacy against gastric diseases and are often associated with severe side effects [5]. Thus, there is an urgent need to identify more effective and safe antiulcer agents. In this context, the use of medicinal plants for the prevention and treatment of different pathologies is in continuous expansion worldwide [6]. Natural products are gaining space and importance in the pharmaceutical industry as well as inspiring the search for new potential sources of bioactive molecules [7]. Herbs, medicinal plants, spices, vegetables, and crude drug substances are considered to be a potential source to combat various diseases including gastric ulcer. In the scientific literature, a large number of medicinal plants with gastric antiulcer potential have been reported [8–10].

*Jasminum sambac*. Linn (Oleaceae) is commonly known as Jasmine. Traditionally, *J. sambac* is used as the treatment of various illnesses such as rheumatism, paralysis, gallstones, and diabetes mellitus. Flowers of *J. sambac* are used to reduce fever, swollen eyes, and bee stings. Leaf part is usually used to reduce the shortness of breath and as treatment of acne. The root part is used to treat headache and insomnia and is believed can accelerate fracture healing. Essential oil of *J. sambac* is used as fragrance for skin care products as it tones the skin as well as reduces skin inflammation [11]. The *J. sambac *flowers and leaves are largely used in folk medicine to prevent and treat breast cancer. Flowers of *J. sambac *are useful to women when brewed as a tonic as it aids in preventing breast cancer and stopping uterine bleeding [12]. Previous studies done on *J. sambac* reveal that the plant antifungal [13] and anti-cancer [14] works. Essential oil and methanol extract from *Jasminum sambac *have *in vitro* antimicrobial and antioxidant activities which could support the use of the plant by traditional healers to treat various infective diseases [11]. Phytochemical studies showed that the roots contain dotriacontanoic acid, dotriacontanol, oleanolic acid, daucosterol, and hesperidin [15]. Ethyl acetate and water extract of leaves of *Jasminum sambac *showed reduction in plasma glucose level, lipid profile, and serum urea in diabetic rats [16]. However, there is no data reported on antiulcer activities within the plant. Hence, the current study was undertaken to evaluate the gastroprotective effects of ethanolic extracts of this plant against HCl/ethanol-induced gastric ulcers in rats and the effect of acidified ethanol and *J. sambac* extract treatment on Hsp70 and Bax proteins in immunohistochemical staining, and on antioxidant status of gastric tissue homogenate they were assessed by determining MDA levels and antioxidants, PGE_2_ and SOD.

## 2. Materials and Methods

### 2.1. Omeprazole

 In this study, omeprazole was used as the reference antiulcer drug and was obtained from the University Malaya Medical Centre (UMMC) Pharmacy. The drug was dissolved in 0.5% w/v carboxymethylcellulose (CMC) and administered orally to the rats in concentrations of 20 mg/kg body weight (5 mL/kg) according to the recommendation of Mahmood et al. [9].

### 2.2. Plant Specimen and Preparation of Extraction

 Fresh *J. sambac* leaf were obtained from Ethno Resources Sdn Bhd, Selangor, Malaysia, and identified by comparison with the Voucher specimen deposited at the Herbarium of Rimba Ilmu, Institute of Science Biology, University of Malaya, Kuala Lumpur. The dried leaves were powdered using electrical blender. Hundred grams of the fine powder were soaked in 500 mL of 95% ethanol in conical flask for 3 days. After 3 days, the mixture was filtered using a fine muslin cloth followed by filter paper (Whatman no. 1) and distilled under reduced pressure in an Eyela rotary evaporator (Sigma-Aldrich, USA) and yielded approximately 11.3%. The dry extract was then dissolved in CMC and administered orally to rats at doses of 62.5, 125, 250, and 500 mg/kg body weight (5 mL/kg body weight) [17].

### 2.3. Acute Toxicity Test and Experimental Animals

 Adult healthy male and female Sprague Dawley rats (6–8 weeks old) were obtained from the Animal house, Faculty of Medicine, University of Malaya, Kuala Lumpur (Ethic no. PM/27/07/2010/MAA (R)). The rats weighted between 150–180 g. The animals were given standard rat pellets and tap water. The acute toxicity study was used to determine a safe dose for the extract. Thirty six Sprague Dawley rats (18 males and 18 females) were randomly assigned equally each into 3 groups labeled as vehicle (CMC, 0.5% w/v, 5 mL/kg), 2 g/kg, and 5 g/kg of *J. sambac* extract preparation, respectively [18] The animals were fasted overnight (food but not water) prior to dosing. Food was withheld for a further 3 to 4 hours after dosing. The animals were observed for 30 min and 2, 4, 8, 24 and 48 h after the administration for the onset of clinical or toxicological symptoms. Mortality, if any, was observed over a period of 2 weeks. The animals were sacrificed on the 15th day. Histology, hematological, and serum biochemical parameters were determined according to the OECD [18]. The study was approved by the ethics Committee for animal experimentation, Faculty of Medicine, University of Malaya, Malaysia. Throughout the experiments, all animals received human care according to the criteria outlined in the Guid for the Care and Use of laboratory Animals prepared by the National Academy of Sciences and published by the national Institute of health.

### 2.4. Experimental Animals for Gastric Ulcer

 Sprague Dawley healthy adult male rats were obtained from the Experimental Animal House, Faculty of Medicine, University of Malaya, and Ethic Number PM/12/05/2010/MAA (R). The rats were divided randomly into 7 groups of 6 rats each. Each rat that weighed between 225–250 g was placed individually in a separate cage (one rat per cage) with wide-mesh wire bottoms to prevent coprophagia during the experiment. The animals were maintained on standard pellet diet and tap water. The study was approved by the Ethics Committee for Animal Experimentation, Faculty of Medicine, University of Malaya, Malaysia.

### 2.5. Gastric Ulcer Induction by HCl/Ethanol

The rats fasted for 24 hours but were allowed free access to drinking water up till 2 hours before the experiment. A gastric injury model based upon a modification of the method described by Mizui and Doteuchi [19] was induced by acidified ethanol solution (150 mM HCl/absolute ethanol) 40 : 60 v/v, (HCl/ethanol solution). Normal control groups were orally administered vehicle CMC (5 mL/kg). Ulcer control groups were orally administered vehicle CMC (5 mL/kg). Reference group received oral doses of 20 mg/kg omeprazole. Experimental groups were orally administered with ethanolic extract of *J. sambac *leaves at doses of 62.5, 125, 250, and 500 mg/kg. One hour after this pretreatment, normal control group was orally administered with CMC, and HCl/ethanol solution (5 mL/kg) was orally administered to ulcer control group, reference group, and experimental group in order to induce gastric ulcers. The rats were euthanized 60 minutes later [10] under an overdose of xylazine and ketamine anesthesia and their stomachs were immediately excised.

### 2.6. Measurement of Acid Content of Gastric Juice (pH)

 Samples of gastric contents were analyzed for hydrogen ion concentration by pH metric titration with 0.1 N NaOH solutions using digital pH meter [20].

### 2.7. Gastric Wall Mucus Determination

The glandular segments of the stomach were removed, weighed, and assessed to determine gastric wall mucus in rats [21]. Each segment was transferred immediately to a 1% Alcian blue solution (in sucrose solution, buffered with sodium acetate at pH 5), and the excess dye was removed by rinsing with sucrose solution. The dye complexes with the gastric wall mucus were extracted with magnesium chloride solution. A 4 mL aliquot of blue extract was then shaken with an equal volume of diethyl ether. The resulting emulsion was centrifuged and the absorbance of the aqueous layer was recorded at 580 nm. The quantity of Alcian blue extracted per gram of glandular tissue (net) was then calculated.

### 2.8. Gross Gastric Lesions Evaluation

 Ulcers of the gastric mucosa appear as elongated bands of black hemorrhagic lesions parallel to the long axis of the stomach. Gastric mucosa of each rat was thus examined for damage. The length and width of the ulcer (mm) were measured by a planimeter (10 × 10 mm^2^ = ulcer area) under dissecting microscope (1.8x). The ulcerated area was measured by counting the number of small squares, 2 mm × 2 mm, covering the length and width of each ulcer band. The sum of the areas of all lesions for each stomach was applied in the calculation of the ulcer area (UA) where in the sum of small squares × 4 × 1.8 = UA (mm^2^) according to the recommendation of Mahmood et al., 2011 [17]. The inhibition percentage (I.0%) was calculated by the following formula according to the recommendation of Mahmood et al. [9]:

(1) $$\begin{array}{cc} \left(\text{I\%}\right) & =\left[\left({\text{UA}}_{\text{control}}\;-\;{\text{UA}}_{\text{treated}}\right)\;÷\;{\text{UA}}_{\text{control}}\right]\\ & \;\times 100\text{\%}. \end{array}$$

### 2.9. Histological Evaluation of Gastric Lesions

Specimens of the gastric walls of each rat were fixed in 10% buffered formalin and processed in a paraffin tissue processing machine. Sections of the stomach were made at a thickness of 5 *μ* and stained with Hematoxylin and eosin for histological evaluation [8, 20].

### 2.10. Immunohistochemistry

Tissue section slides were heated at 60°C for approximately 25 min in hot air oven (Venticell, MMM, Einrichtungen, Germany). The tissue sections were deparaffinized in xylene and rehydrated using graded alcohol. Antigen retrieval process was performed in 10 mM sodium citrate buffer boiled in microwave. Immunohistochemical staining was conducted according to manufacturer's protocol (Dakocytomation, USA). Briefly, endogenous peroxidase was blocked by peroxidase block (0.03% hydrogen peroxide containing sodium azide) for 5 min. Tissue sections were washed gently with wash buffer, and then incubated with HSP70 (1 : 500) and Bax (1 : 200) biotinylated primary antibodies for 15 min. The sections were rinsed gently with wash buffer and placed in buffer bath. The slides were then placed in a humidified chamber and sufficient amount of streptavidin-HRP (streptavidin conjugated to horseradish peroxidase in PBS containing an antimicrobial agent) was added and incubated for 15 min. Then, tissue sections were rinsed gently in wash buffer and place in buffer bath. Diaminobenzidined (DAB-) substrate-chromagen was added to the tissue sections and incubated further for 5 min following washing and counterstaining with hematoxylin for 5 second. The sections were then dipped in weak ammonia (0.037 mol/L) 10 times and then rinsed with distilled water and cover slipped. Positive findings of the immunohistochemical staining should be seen as brown stains under light microscope.

### 2.11. Antioxidant Activity of Gastric Homogenate

#### 2.11.1. Sample Preparations

For assays of PGE_2_, SOD and MDA in gastric tissue of analyzed groups, the tissue homogenates were produced. All the processes were handled at 4°C throughout. Gastric tissue was cut into three small pieces and then weighed (about 200 mg for each) [22]. The tissues were homogenized in teflon homogenizer (Polytron, Heidolph RZR 1, Germany) using the appropriate buffer, depending upon the variable to be measured. After centrifugation at 18,000 ×g for 15 min at 4°C, the supernatant was extracted.

#### 2.11.2. Measurement of Superoxide Dismutase (SOD) Activity

SOD activity was measured according to Sun et al. [23]. The activity of the enzyme was evaluated by measuring its capacity to inhibit the photochemical reduction of nitro-blue tetrazolium (NBT). In this assay, the photochemical reduction of riboflavin generates O^2−^ that reduces the NBT to produce formazan salt, which absorbs at a wavelength of 560 nm. In the presence of SOD, the reduction of the NBT is inhibited because the enzyme converts the superoxide radical to peroxide. The results are expressed as the quantity of SOD necessary to inhibit the rate of reduction of the NBT by 50% in units of the enzyme per gram of protein. Homogenates (10% of tissue in phosphate buffer) were centrifuged (10 minutes, 3,600 rpm, 4°C), and the supernatant was removed and centrifuged a second time (20 minutes, 12,000 rpm, 4°C). The resulting supernatant was assayed. In a dark chamber, 1 mL of the reactant (50 mM phosphate buffer, 100 nM EDTA, and 13 mM l-methionine, pH 7.8) was mixed with 30 *μ*L of the sample, 150 *μ*L of 75 *μ*M NBT, and 300 *μ*L of 2 *μ*M riboflavin. The tubes containing the resulting solution were exposed to fluorescent light bulbs (15 W) for 15 minutes and then read using a spectrophotometer at 560 nm.

#### 2.11.3. Measurement of Membrane Lipids Peroxidation (MDA)

The rate of lipoperoxidation in the gastric mucous membrane was estimated by determination of malondialdehyde (MDA) using the thiobarbituric acid reactive substances (TBARSs) test. The stomaches were washed with phosphate buffered saline to minimize the interference of hemoglobin with free radicals and to remove blood adhered to the mucous membrane. The stomaches were homogenized with 10% of the tissue with potassium phosphate buffer. Then, 250 *μ*L was removed and stored at 37°C for 1 hour, after which 400 *μ*L of 35% perchloric acid was added, and the mixture was centrifuged at 14,000 rpm for 20 minutes at 4°C. The supernatant was removed, mixed with 400 *μ*L of 0.6% thiobarbituric acid, and incubated at 95–100°C for 1 hour. After cooling, the absorbance at 532 nm was measured. A standard curve was generated using 1,1,3,3-tetramethoxypropane. The results were expressed as nmol of MDA/mg of protein. The concentration of the proteins was measured using the method described by Bradford method [24]. Measurement of total protein in the stomach sample after ethanol-induced lesions was done, the method is based on the interaction of the Coomassie Blue G250 dye with proteins. At the pH of the reaction, the interaction between proteins of high molecular weight and the dye causes a shift in the dye to the anionic form, which absorbs strongly at 595 nm. Solutions of albumin standard, distilled water, buffer, and each sample were added to the wells. For sample preparation, 2 *μ*L of a sample and 38 *μ*L of buffer were added to each well. Then, 200 *μ*L Bradford's solutions (diluted 5×) were added to each well. After 5 minutes, a reading was taken at a wavelength of 595 nm [24].

#### 2.11.4. Measurement of PGE2 Formation Using Enzyme Immunoassays

The gastric mucosa was weighed, minced with scissors, and homogenized at 48°C in PBS buffer. Homogenates were centrifuged at 13 400 g for 10 min, and the supernatants were subjected to a PGE2 assay using a PGE2 Monoclonal Enzyme Immunoassay Kit (Sigma-Aldrich, Malaysia).

### 2.12. Statistical Analysis

All values were reported as mean ± S.E.M. The statistical significance of differences between groups was assessed using one-way ANOVA. A value of *P* < 0.05 was considered significant.

## 3. Results

### 3.1. Acute Toxicity Study

**Figure 1:**
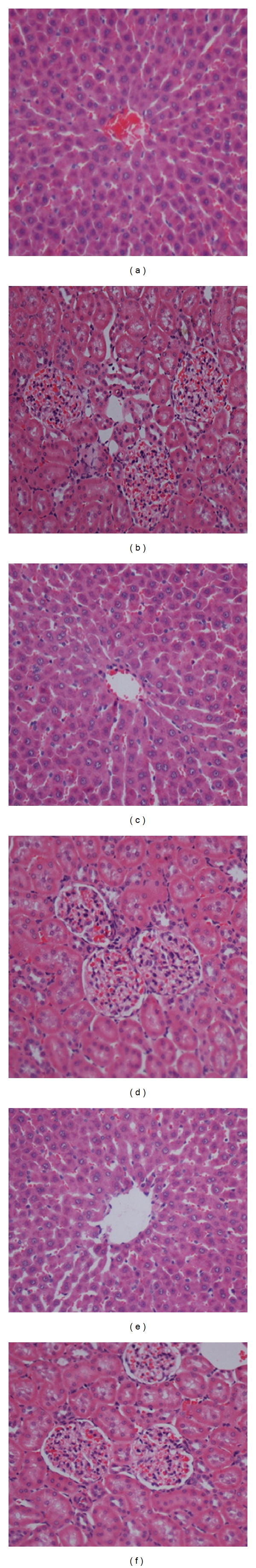
Histological sections of the liver and kidney from the acute toxicity test. (a and b) Rats treated with 5 mL/kg of the vehicle (CMC). (c and d) Rats treated with 2000 mg/kg (5 mL/kg) of the *J. sambac* extract. (e and f) Rats treated with 5000 mg/kg (5 mL/kg) of the *J. sambac* extract. There is no significant difference in the structures of the livers and kidneys between the treated and control groups (Hematoxylin and Eosin stain, 20× magnifications).

Acute toxicity is a study in which the animals were treated with the *J. sambac* extract at a dose of 2 g/kg and 5 g/kg and were kept under observation for 14 days. All the animals remained alive and did not manifest any significant visible toxicity at these doses. Thus, clinical observations, serum biochemistry, and histopathology data did not show any significant differences between control and treated groups (Figure 1 and Table 1).

### 3.2. Gross Evaluation of Gastric Lesions

**Figure 2:**
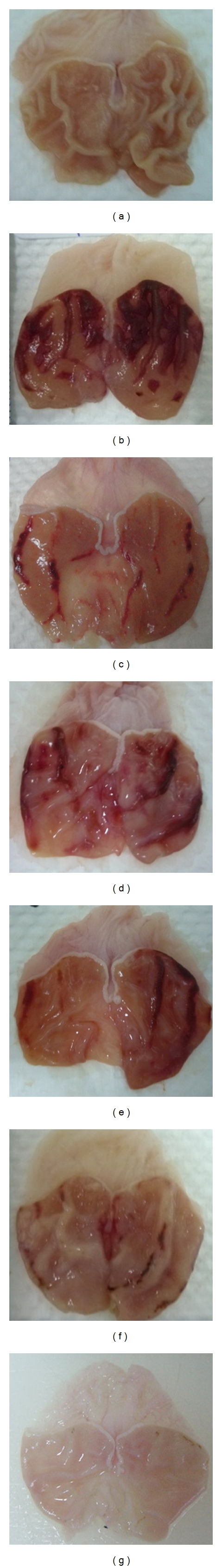
Gross appearance of the gastric mucosa in rats. (a) Rats pretreated with 5 mL/kg CMC (normal control). No injuries to the gastric mucosa are seen. (b) Rats pretreated with 5 mL/kg CMC (ulcer control). Severe injuries are seen in the gastric mucosa. HCl/ethanol produced extensive visible hemorrhagic necrosis of gastric mucosa. (c) Rats pretreated with omeprazole (20 mg/kg). Injuries to the gastric mucosa are milder compared to the injuries seen in the ulcer control rats. (d). Rat pretreated with *J. sambac *extract (62.50 mg/kg). Moderate injuries are seen in the gastric mucosa. The extract reduces the formation of gastric lesions induced by acidified ethanol. (e). Rat pretreated with *J. sambac *extract (125 mg/kg). Moderate injuries are seen in the gastric mucosa. The extract reduces the formation of gastric lesions induced by acidified ethanol (f). Rat pretreated with *J. sambac *extract (250 mg/kg). Mild injuries are seen in the gastric mucosa. The extract reduces the formation of gastric lesions induced by acidified ethanol. (g) rats pretreated with 500 mg/kg of *J. sambac* extract. No injuries to the gastric mucosa are seen instead flattening of gastric mucosa is seen.

**Figure 3:**
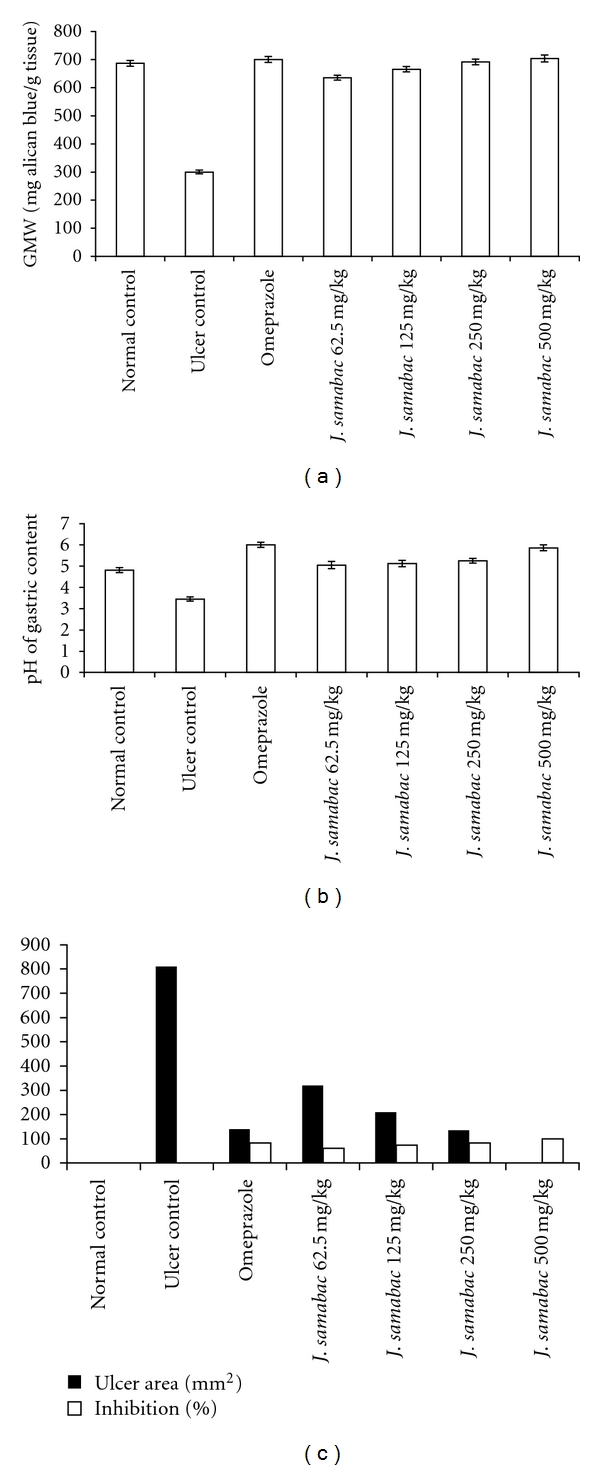
(a) Effect of *J. sambac* on GMW. (b) Effect of *J. sambac* on pH of gastric content. (c) Effect of *J. sambac* on Ulcer area and inhibition %.

The antiulcer activity of *J. sambac *leaf extract in HCl/ethanol-induced gastric lesion model is shown in Figures 2 and 3. Results showed that rats pretreated with omeprazole or *J. sambac *extracts before being given HCl/ethanol solution had significantly reduced areas of gastric ulcer formation compared with ulcer control group (Figures 2 and 3). Acidified ethanol solution produced extensive visible black hemorrhagic lesions of gastric mucosa. Moreover, this plant extract significantly suppressed the formation of the ulcers and it was interesting to note the flattening of gastric mucosal folds in rats pretreated with the extract of this plant (500 mg/kg). It was also observed that protection of gastric mucosa was the most prominent in rats pretreated with 500 mg/kg leaf extract (Figures 2 and 3). The significant inhibition of gastric ulcer in rats pretreated with *J. sambac* extract (250 mg/kg) was comparable with omeprazole which is a standard drug used for curing gastric ulcer (Figures 2 and 3).

### 3.3. Gastric Wall Mucosal Evaluation

Treatment with HCl/ethanol caused a significant decrease in the mucus content of the gastric wall in the untreated animals (ulcer control group, Figure 3). The depleted gastric mucus was significantly replenished after pretreatment with the *J. sambac* extract. It was also found that the pretreatment with *J. sambac* at doses of 62.5, 125, 250, and 500 mg/kg significantly increased the amount of gastric mucus in the acidified ethanol-ulcerated rats (Figure 3).

### 3.4. pH of Gastric Content

The acidity of gastric content in experimental animals pretreated with omeprazole or *J. sambac* leaf was decreased significantly compared with that of the ulcer control group (*P* < 0.05, Figure 3).

### 3.5. Histological Evaluation of Gastric Lesions

**Figure 4:**
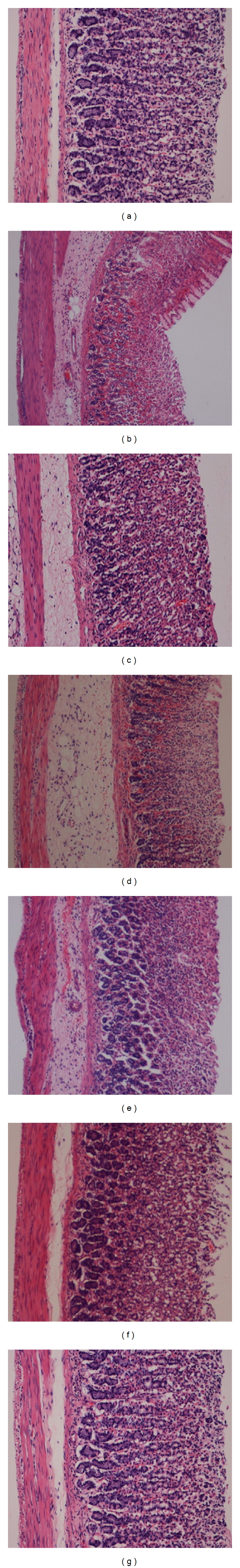
Histological study of HCl/ethanol-induced gastric mucosal damage in rats. (a) Rats pretreated with 5 mL/kg of CMC (Normal control group). No injuries to the gastric mucosa are seen. (b) Rats pretreated with 5 mL/kg of CMC (ulcer control group). There is severe disruption to the surface epithelium and necrotic lesions penetrating deeply into mucosa and extensive edema of submucosa layer and leucocytes infiltration is present. (c) Rats pretreated with omeprazole (20 mg/kg). Mild disruption of the surface epithelium mucosa is seen. There is edema and leucocytes infiltration of the submucosal layer. (d) Rat pretreated with *J. sambac *extract (62.50 mg/kg). Moderate disruption of surface epithelium is present. There is submucosal edema and leucocytes infiltration. (e) Rats pretreated with *J. sambac *extract (125 mg/kg). There is mild disruption to the surface epithelium. There is edema with leucocytes infiltration of the submucosal layer. (f). Rats pretreated with *J. sambac *extract (250 mg/kg). There is mild disruption to the surface epithelium. There is no edema or leucocytes infiltration of the submucosal layer. (g) Rats pretreated with *J. sambac *extract (500 mg/kg). There is no disruption to the surface epitheliumand no edema or leucocytes infiltration of the submucosal layer (H&E stain 10×).

Histological observation of HCl/ethanol-induced gastric lesions in ulcer control group showed comparatively extensive damage to the gastric mucosa and necrotic lesions penetrate deeply into mucosa and extensive oedema and leucocytes infiltration of the submucosal layer are present (Figure 4). Rats that received pretreatment with *J. Sambac* extract had comparatively better protection of the gastric mucosa as seen by reduction of ulcer area, reduced submucosal oedema, and leucocytes infiltration (Figure 4). This plant has been shown to exert the cytoprotective effects in a dose-dependent manner.

### 3.6. Immunohistochemistry

**Figure 5:**
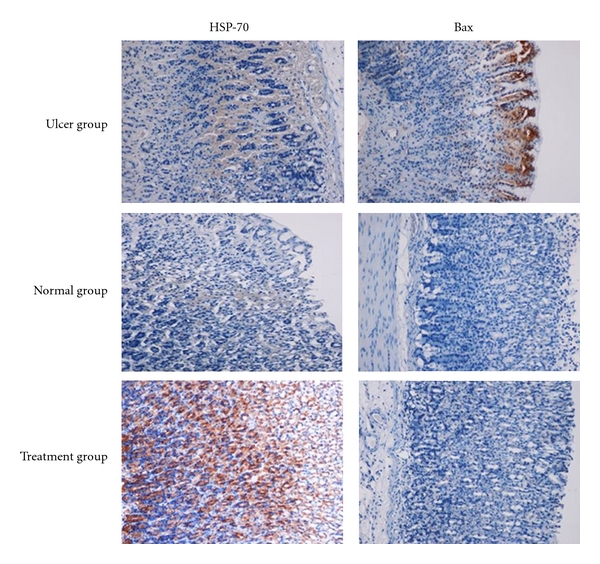
Immunohistochemical analysis of expression of Hsp and Bax proteins in the stomach of rats in HCl/ethanol-induced gastric ulcer. Immunohistochemistry staining of Hsp70 and Bax proteins showed overexpression of Hsp70 protein and downexpression of Bax protein in rats pretreated with plant extract (magnification 10×).

Immunohistochemical results demonstrated that treatment of HCl/ethanol induced injury rats with *J. Sambac* extract cause over-expression of Hsp70 protein (Figure 5). In addition to this, the expression of HSP70 protein in normal control and HCl/ethanol-induced gastric tissues (ulcer control group) was found to be downregulated compared to Hsp70 expression in *J. Sambac* extract-treated group (Figure 5). Immunohistochemical staining of Bax protein demonstrated that pretreatment of HCl/ethanol-induced injury rats with *J. Sambac* extract cause downexpression of Bax protein (Figure 5). In addition to this, the expression of Bax in HCl/ethanol-induced gastric tissues (ulcer control group) was found to be up-regulated compared to *J. Sambac*-treated group (Figure 5).

### 3.7. Enzymatic Activities in the Gastric Tissue Homogenate

**Figure 6:**
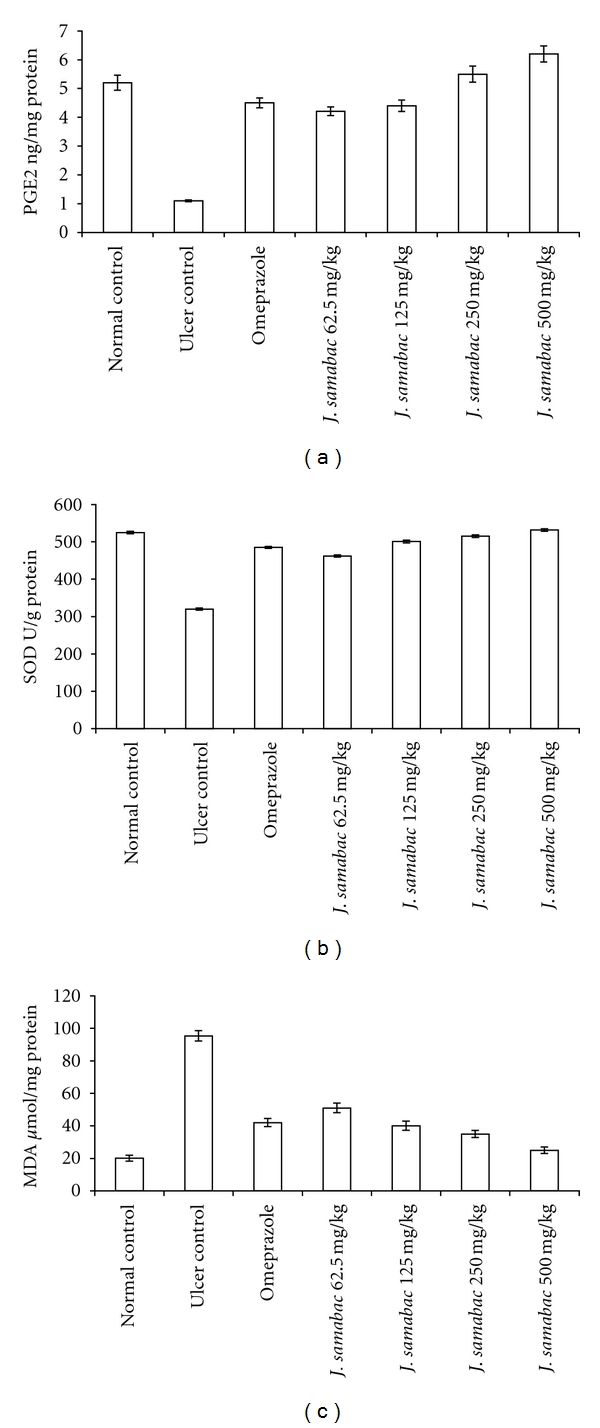
(a) Effect of *J. sambac *leaf extract on PGE_2_ in gastric tissue homogenate. (b) Effect of *J. sambac *leaf extract on SOD in gastric tissue homogenate. (c) Effect of *J. sambac *leaf extract on MDA in gastric tissue homogenate.

In gastric tissue homogenate, both PGE_2_ and SOD activities in ulcer control group were significantly lower compared with normal control group (Figure 6). Administration of omeprazole or *J. sambac* before HCl/ethanol significantly rise the PGE_2_ and SOD compared with ulcer control group (Figure 6). Administration of HCl/ethanol significantly increase the MDA level of gastric homogenate in ulcer control group compared with normal control. Administration of omeprazole or *J. sambac *extract decreased the MDA level in gastric tissue compared with ulcer control group (Figure 6).

## 4. Discussion

 Peptic ulcers are caused by an imbalance between the protective and the aggressive mechanisms of the mucosa, and are the result of the association of several endogenous factors and aggressive exogenous factors that are related to living conditions [25]. In the HCl/ethanol-induced gastric ulceration model, HCl causes severe damage to gastric mucosa [26], whereas ethanol produces necrotic lesions by direct necrotizing action which in turn reduces defensive factors like the secretion of bicarbonate and production of mucus [27]. Ethanol-induced gastric lesions impaired gastric defensive factors such as mucus and mucosa circulation [28]. Ethanol causes necrotic lesions of the gastric mucosa in a multifactorial way. It can reach the mucosa by disruption of the mucus-bicarbonate barrier and cause cell rupture in the wall of blood vessels. These effects are probably due to biological actions, such as of lipid peroxidation, formation of free radicals, intracellular oxidative stress, changes in permeability and depolarization of the mitochondrial membrane prior to cell death [29]. Oral administration of absolute ethanol is noxious to the stomach since it affects the gastric mucosa topically by disrupting its barrier and provoking pronounced microvascular changes within a few minutes after its application [30]. In addition, it produces linear hemorrhagic lesions, extensive submucosal edema, mucosal friability, inflammatory cells infiltration, and epithelial cell loss in the stomach, which are typical characteristics of alcohol injury [31]. The pathogenesis of ethanol-induced gastric mucosal damage occurs directly and indirectly through various mediators such lipoxygenase, cytokines, and oxygen-derived free radicals [32]. Mucus secretion is regarded as a crucial defensive factor in the protection of the gastric mucosa from gastric lesions [33].

 In the present study, acute toxicity test did not show any signs of toxicity and mortality. Behavioural changes like irritation, restlessness, respiratory distress, abnormal locomotion, and catalepsy over a period of 14 days were not observed. This revealed that the plant is safe and has no toxicity when administered orally up to 5 g/kg. Although ulcer etiology is unknown in most cases, it is generally accepted that an imbalance between acid and pepsin production and mucosal integrity would be causative factor acting via endogenous defense mechanisms. The experimental results of the study showed that *J. sambac* extract has an effective antisecretory and antiulcer activity against ethanol-induced gastric mucosal injury. The plant extract decreased the acidity and increased the gastric wall mucus is consistence with results reported by Al-Attar [34]. Similarly, Mahmood et al. [35] discovered a reduction in gastric acidity in treated animals. Pretreatment with *J. sambac* extract could partly reduce the ulcer area and prevent gastric ulceration. Omeprazole exhibits an antisecretory and protective effect [36]. In ulcer control group, it increased the acid secretion resulting into an increase in ulcer area [37]. Omeprazole, as a proton pump inhibitor (PPI), offered a fairly protected gastric mucosa and has been widely used as an acid inhibitor agent for the treatment of disorders related to gastric acid secretion [38]. PPIs are capable of producing almost complete suppression of acid secretion. The mechanism of action of omeprazole is such that it binds very specifically to a single subunit of the H^+^, K^+^-ATpase at the secretory surface of parietal cell and inactivates it, and it reduces acid secretion regardless of the source of secretory stimulation. Omeprazole are effective in treating peptic ulcer disease and gastroesophageal reflux with both short- and long-term use [39]. The pathogenesis of mucosal damage in the stomach includes the generation of reactive oxygen species (ROS) that seem to play a vital role in the formation of lipid peroxides, accompanied by impairment of antioxidative enzyme activity of cells [2].

Oxidative stress plays important role in the pathogenesis of various diseases including gastric ulcer, with antioxidants being reported to play a significant role in protection of gastric mucosa against various necrotic agents [40]. Antioxidants could help to protect cells from damage caused by oxidative stress and enhanced the body's defense systems against degenerative diseases. Administration of antioxidants inhibits ethanol-induced gastric injury in rat [41]. *J. sambac* extracts have been shown to contain antioxidants [11] and it is likely that gastroprotective activity exerted by this plant extract could be attributed to its antioxidant property. In addition, *J. sambac* extract are reported to contain flavonoids [42]. Histopathology results of the present study also revealed protection of gastric mucosa and inhibition of leucocytes infiltration of gastric wall in rats pretreated with *J. sambac* extract. Activation and infiltration of neutrophils appear to be involved in the initial processes of formation of the lesion. Similarly, Abdulla et al. [8] demonstrated that the reduction of neutrophil infiltration into ulcerated gastric tissue promotes the prevention of gastric ulcers in rats. Wasman et al. [10] showed that oral administration of plant extract before ethanol administration significantly decreased neutrophil infiltration of gastric mucosa. Absolute alcohol would extensively damage the gastric mucosa leading to increased neutrophil infiltration into the gastric mucosa. Oxygen free radicals derived from infiltrated neutrophils in ulcerated gastric tissues have inhibitory effect on gastric ulcers healing in rats. Neutrophils are a major source of inflammatory mediators and can release potent reactive oxygen species such as superoxide, hydrogen peroxide, and myeloperoxidase derived oxidants as a result they mediate lipid peroxidation [43]. These reactive oxygen species are highly cytotoxic and can induce tissue damage [44]. It is speculated that the gastroprotective effect exerted by this plant extract could be attributed to its anti-inflammatory activity as proved previously [11]. This anti-inflammatory activity could also be a key factor in the prevention of gastric ulcer as reported by Swarnakar et al. [45].

Excessive production of myeloperoxidase (MPO) that exists in neutrophil leukocyte cells and catalyses the formation of toxic hypochlorous acid (HOCl) from hydrogen peroxide causes cell membrane damage by lipid peroxidation. MDA is the final product of lipid peroxidation and is used to determine lipid peroxidation levels in tissues [46]. Gastric MDAs were increased by ulcer control group and decreased by *J. sambac* extract administration, another indicator of a possible antioxidant activity of this plant.

Studies have shown that the excessive recruitment and metabolic activation of neutrophils generate free radicals in several models of gastric damage resulting in inflammation-dependent tissue damage [47]. In the present study, we observed flattening of the mucosal folds which suggests that gastroprotective effect of *J. sambac* leaf extract might be due to a decrease in gastric motility. It is reported that the changes in the gastric motility may play a role in the development and prevention of experimental gastric lesions [8]. Relaxation of circular muscles may protect the gastric mucosa through flattening of the folds. This will increase the mucosal area exposed to necrotizing agents and reduce the volume of the gastric irritants on rugal crest [8, 10]. Ethanol produces a marked contraction of the circular muscles of rat fundic strip. Such a contraction can lead to mucosal compression at the site of the greatest mechanical stress, at the crests of mucosal folds leading to necrosis and ulceration [17]. Gastric tissue homogenate from animals pretreated with omeprazole or plant extract showed significant antioxidant activity by decreasing the levels of MDA and by elevating the levels of PGE_2_ and SOD in response to oxidative stress due to absolute ethanol administration. Free radicals and reactive oxygen species (ROS) that are continuously produced in human body are the cause of cell damage. Therefore, tissues must be protected from oxidative injury through intracellular as well as extracellular antioxidants [48]. SOD converts superoxide to hydrogen peroxide (H_2_O_2_) which is then transformed into water by catalase in lysosomes or by glutathione peroxidase (GPx) in mitochondria [49]. SOD-mediated catalysis of superoxide radical anion (O_2_^∙−^) into less noxious hydrogen peroxide (H_2_O_2_) represents the first line of antioxidant defense. In our study, SOD activities were significantly reduced after ethanol administration in ulcer control group, and this reduction was prevented by pretreatment with *J. sambac* leaf extract. Reduced activities of SOD in gastric tissue homogenate in ulcer control group that have been observed in our study may be due to increased production of reactive oxygen radicals that can themselves reduce the activity of these enzymes [50]. The reduction of these enzymes in gastric tissue homogenate may lead to a number of deleterious effects. Any compound, natural or synthetic with antioxidant activities might contribute towards the total/partial alleviation of such damage. Lipid peroxidation was found to be an important pathophysiological event in a variety of diseases including gastric ulcer [51]. It is well known that MDA from lipid peroxidation reacts with DNA bases and induces mutagenic lesions [52]. Pratibha et al. [53] showed that the activated oxygen species can in turn induce cellular events such as enzyme inactivation, DNA strands cleavage and also membrane lipid peroxidation. Conclusion, *J. sambac *play a protective role against gastric ulcer. Its antiulcer effect is related to increasing secretion of adherent mucus and pH of gastric content, which may inhibit generation of oxygen-derived free radicals, and decrease the consumption of SOD and maintain content of MDA at normal level.

Prostaglandin E_2_ (PGE_2_) plays an important role in the regulation of gastric mucus secretion. PGE_2_ has protective effects against various gastric injury models [54, 55]. Ethanol has been shown to reduce the mucosal PGE_2_ content [56]. PGE_2_ is the most abundant gastrointestinal prostaglandin and it regulates functions of the gut, including motility and secretion [57]. PGE_2_ has also been shown to exert a protective action on the stomach through the activation of EP receptors [58]. The role of PGE_2_ in mediating the gastroprotective effect of *J. sambac *was investigated. The results of the present study suggest that the gastroprotective effect of *J. sambac *is mediated partially by PGE_2_ as direct measurement of its mucosal level confirmed that its biosynthesis was significantly enhanced by compound. It has been shown that prostaglandins influence virtually every component of the mucosal defense: stimulating mucus and bicarbonate secretion, maintaining mucosal blood flow, enhancing the resistance of epithelial cells to injury induced by cytotoxins, and inhibiting leukocyte recruitment [59]. Additionally, earlier studies show that prostaglandins exert a gastroprotective action against gastric mucosal lesions through maintenance of gastric mucus synthesis and secretion [60].

Hsp70 proteins defend cells from oxidative stress or heat shock. Ethanol-generated ROS normally act to inhibit the expression of HSP and increase the expression of Bx. Hsp70 prevents these partially denatured proteins from aggregating and allows them to refold. The overexpression of HSP70 noticed in this study could suggest that the plant extract protected the gastric tissues through the upregulation of Hsp70.

HSP70 is a 70 kDa protein from the HSP family present on mammalian cells. It is the most conserved and abundantly produced protein in response to different forms of stress [61], such as heat, toxic agents, infection, and proliferation [62]. These proteins are responsible to protect cellular homeostatic processes from environmental and physiologic injuries by preserving the structure of normal proteins and repairing or removing damaged proteins [63], which makes the study of this protein an interesting element for possible mechanisms of action elucidation. Our results show significant expression of HSP70 in pretreated plant extract. The HSP70 family functions as a molecular chaperone and reduces stress-induced denaturation and aggregation of intracellular proteins. In addition to its chaperoning activities, Hsp70 has been suggested to exert its cytoprotective action by protecting mitochondria and by interfering with the stress-induced apoptotic program [64].

Tomisato et al. [65] showed the evidence of adaptive cytoprotection through HSP70 induction in animal model experiment that pretreatment of ethanol, which induced Hsp70, made cell to indomethacin injury, and Jin et al. [66] showed that HSP70 could play important role in gastric mucosal adaptation when the PGE_2_ level is suppressed by NSAID. Oyake et al. [67] added the data overexpression of Hsp70 confers protection against monochloramine-induced gastric mucosal injury.

Several publications that Hsp70 inductions improved both short-term survival 2-fold and long-term survival 5-fold in mice challenged with ethanol and endotoxin in mice [68]. Hsp70 inductions protected rats against ethanol-induced gastric mucosal damages [69], and Hsp70 inductions led to inactivation of MAPK in alcohol-induced gastric injuries [70] all raised the possibility of the intervention of phytoceuticals as novel therapeutics for preventing alcohol-associated gastric damages.

## 5. Conclusions

In conclusion, an acute toxicity study demonstrated that rats treated with the *J. sambac* (2000 mg or 5000 mg/kg) manifested no abnormal signs. This plant could significantly protect the gastric mucosa against ethanol-induced injury. Such protection was ascertained grossly by significant increase in the gastric wall mucus in comparison with the ulcer control group. Also reduction of ulcer areas in the gastric wall as well as by the reduction or inhibition of edema and leukocytes infiltration of the submucosal layers were shown histologically. Immunohistochemistry staining of Hsp70 and Bax proteins showed overexpression of Hsp70 protein and downexpression of Bax protein in rats pretreated with plant extract. Assays of PGE2, SOD and MDA levels of gastric tissue homogenates reveal that this plant significantly increases the PGE2 and SOD and decreased the level of lipid peroxidation (MDA) in the treated group compared with the ulcer control group. This study provides evidence that the *J. sambac *possesses an antigastric ulcer effect, which is related partly to a preservation of gastric mucus secretion, to increased production of HSP70 protein, and to the antioxidant enzymes.

## Figures and Tables

**Table 1a:** (a)

Dose	Sodium (mmol/L)	Pottasium (mmol/L)	Chloride (mmol/L)	CO_2_ (mmol/L)	Anion gap (mmol/L)	Urea (mmol/L)	Creatinine (*μ*mol/L)
Vehicle (CMC)	141.33 ± 0.58	4.98 ± 0.04	103.88 ± 0.82	24.12 ± 0.54	18.75 ± 0.46	5.45 ± 0.45	34.83 ± 2.17
LD (2 g/kg)	142.05 ± 0.55	5.02 ± 0.09	105.29 ± 1.02	22.67 ± 0.81	18.55 ± 0.62	6.06 ± 0.83	33.87 ± 2.29
HD (5 g/kg)	143.14 ± 0.68	4.91 ± 0.06	104.35 ± 0.54	23.90 ± 0.64	19.15 ± 0.45	5.63 ± 0.37	35.05 ± 2.26

**Table 1b:** (b)

Dose	Sodium (mmol/L)	Pottasium (mmol/L)	Chloride (mmol/L)	CO_2_ (mmol/L)	Anion gap (mmol/L)	Urea (mmol/L)	Creatinine (*μ*mol/L)
Vehicle (CMC)	141.87 ± 0.42	4.83 ± 0.14	105.78 ± 0.67	23.33 ± 0.41	18.00 ± 0.25	7.95 ± 0.33	41.76 ± 2.75
LD (2 g/kg)	142.07 ± 0.56	4.55 ± 0.16	105.85 ± 0.65	22.65 ± 0.42	17.49 ± 0.46	7.97 ± 0.49	42.00 ± 2.36
HD (5 g/kg)	142.15 ± 0.47	4.63 ± 0.18	107.03 ± 0.53	21.96 ± 0.75	17.67 ± 0.48	8.31 ± 0.68	43.13 ± 2.24

Values expressed as mean ± S.E.M. There are no significant differences between groups. Significant value at *P* < 0.05.

**Table 1c:** (c)

Dose	Total protein (g/L)	Albumin (g/L)	Globulin (g/L)	TB (*μ*mol/L)	CB (*μ*mol/L)	AP (IU/L)	ALT (IU/L)	AST (IU/L)	GGT (IU/L)
Vehicle (CMC)	60.45 ± 1.25	9.62 ± 0.49	52.02 ± 1.40	2.17 ± 0.17	1.00 ± 0.00	153.00 ± 6.35	50.08 ± 1.62	172.95 ± 6.13	3.26 ± 0.25
LD (2 g/kg)	58.86 ± 0.86	8.81 ± 0.38	50.71 ± 1.21	2.13± 0.16	1.00 ± 0.00	154.17 ± 8.10	48.33 ± 0.58	174.23 ± 5.14	3.65 ± 0.42
HD (5 g/kg)	60.15 ± 1.05	9.17 ± 0.46	50.33 ± 1.24	2.02 ± 0.13	1.00 ± 0.00	155.00 ± 7.04	47.87 ± 1.55	175.15 ± 7.02	3.37 ± 0.18

**Table 1d:** (d)

Dose	Total protein (g/L)	Albumin (g/L)	Globulin (g/L)	TB (*μ*mol/L)	CB (*μ*mol/L)	AP (IU/L)	ALT (IU/L)	AST (IU/L)	GGT (IU/L)
Vehicle (CMC)	64.33 ± 1.26	11.19 ± 0.17	53.17 ± 1.28	2.00 ± 0.00	1.00 ± 0.00	108.83 ± 4.13	43.17 ± 2.91	171.83 ± 6.38	3.67 ± 0.33
LD (2 g/kg)	63.75 ± 1.19	11.05 ± 0.45	52.33 ± 1.26	2.00 ± 0.00	1.00 ± 0.00	98.83 ± 5.25	42.96 ± 2.70	172.17 ± 6.35	3.50 ± 0.51
HD (5 g/kg)	65.02 ± 2.65	11.30 ± 0.43	53.02 ± 1.25	2.00 ± 0.00	1.00 ± 0.00	102.67 ± 5.17	44.02 ± 1.85	174.28 ± 5.26	3.22 ± 0.44

Values expressed as mean ± S.E.M. There are no significant differences between groups. Significant value at *P* < 0.05.

TB: total bilirubin; CB: conjugated bilirubin; AP: alkaline phosphatase; ALT: alanine aminotransferase; AST: aspartate aminotransferase; GGT: G-glutamyl transferase.
